# Population Genetics as a Tool to Select Tsetse Control Strategies: Suppression or Eradication of *Glossina palpalis gambiensis* in the Niayes of Senegal

**DOI:** 10.1371/journal.pntd.0000692

**Published:** 2010-05-25

**Authors:** Philippe Solano, Dramane Kaba, Sophie Ravel, Naomi A. Dyer, Baba Sall, Marc J. B. Vreysen, Momar T. Seck, Heather Darbyshir, Laetitia Gardes, Martin J. Donnelly, Thierry De Meeûs, Jérémy Bouyer

**Affiliations:** 1 Institut de Recherche pour le Développement (IRD)/Centre International de Recherche pour l'Elevage en zones Subhumides (CIRDES), UMR 177 IRD-Centre de coopération Internationale en Recherche Agronomique pour le Développement (CIRAD), CIRDES 01 BP 454, Bobo-Dioulasso, Burkina Faso; 2 Institut Pierre Richet, BP V31, Abidjan, Côte d'Ivoire; 3 IRD, UMR 177 IRD CIRAD, Laboratoire de Recherche et de Contrôle des Trypanosomoses (LRCT) Campus de Baillarguet, Montpellier, France; 4 Vector Group, Liverpool School of Tropical Medicine, Liverpool, United Kingdom; 5 Direction des Services Vétérinaires, Dakar, Sénégal; 6 Entomology Unit, FAO/IAEA Agriculture and Biotechnology Laboratory, Joint FAO/IAEA Programme of Nuclear Techniques in Food and Agriculture, Seibersdorf, Austria; 7 Institut Sénégalais de Recherches Agricoles, Laboratoire National d'Elevage et de Recherches Vétérinaires, Service de Parasitologie, Dakar – Hann, Sénégal; 8 CNRS, Délégation Languedoc-Roussillon, Montpellier, France; 9 Cirad, UMR Contrôle des maladies animales exotiques et émergentes, Campus International de Baillarguet, Montpellier, France; Foundation for Innovative New Diagnostics (FIND), Switzerland

## Abstract

**Background:**

The Government of Senegal has initiated the “Projet de lutte contre les glossines dans les Niayes” to remove the trypanosomosis problem from this area in a sustainable way. Due to past failures to sustainably eradicate *Glossina palpalis gambiensis* from the Niayes area, controversies remain as to the best strategy implement, i.e. “eradication” versus “suppression.” To inform this debate, we used population genetics to measure genetic differentiation between *G. palpalis gambiensis* from the Niayes and those from the southern tsetse belt (Missira).

**Methodology/Principal Findings:**

Three different markers (microsatellite DNA, mitochondrial *CO1* DNA, and geometric morphometrics of the wings) were used on 153 individuals and revealed that the *G. p. gambiensis* populations of the Niayes were genetically isolated from the nearest proximate known population of Missira. The genetic differentiation measured between these two areas (θ = 0.12 using microsatellites) was equivalent to a between-taxa differentiation. We also demonstrated that within the Niayes, the population from Dakar – Hann was isolated from the others and had probably experienced a bottleneck.

**Conclusion/Significance:**

The information presented in this paper leads to the recommendation that an eradication strategy for the Niayes populations is advisable. This kind of study may be repeated in other habitats and for other tsetse species to (i) help decision on appropriate tsetse control strategies and (ii) find other possible discontinuities in tsetse distribution.

## Author Summary

Tsetse flies transmit trypanosomes to humans (sleeping sickness) and animals (nagana). Controlling these vectors is a very efficient way to contain these diseases. There are several strategies and methods that can be used for control, each being more or less efficient depending on several factors. The Government of Senegal wants to sustainably eliminate trypanosomosis from the Niayes region by controlling the tsetse vector, *Glossina palpalis gambiensis*. To reach this objective, two different strategies may be used: suppression (decrease in tsetse densities) or eradication (remove all the tsetse in the region until last one). For eradication, the approach has to be area-wide, i.e. the control effort targets an entire pest population within a circumscribed area, to avoid any possible reinvasion. Three different tools (microsatellite DNA, mitochondrial DNA and morphometrics) were used, and all showed an absence of gene flow between *G. p. gambiensis* from the Niayes and from the nearest known population in the south east of the country (Missira). This genetic isolation of the target population leads to the recommendation that an eradication strategy for the Niayes populations is advisable. This kind of study may be extended to other areas on other tsetse species.

## Introduction

The Niayes of Senegal harbours the most northern and western population of *Glossina palpalis gambiensis* Vanderplank, which is a major vector of the debilitating diseases Human African Trypanosomosis (HAT) or sleeping sickness, and African Animal Trypanosomosis (AAT) or nagana (reviewed in [1]). Particular meteorological and ecological characteristics of this area provide great potential for agricultural development in general and animal production (cattle, donkeys, horses, small ruminants, pigs and poultry) in particular. Most of these animals are however susceptible to AAT which is seriously limiting the development of efficient and productive, sustainable livestock systems. The socio-economic impact of the disease is therefore dramatic and very often underestimated [2]. In the 1970s and 1980s, it was attempted to eliminate the *G. p. gambiensis* population from the Niayes mainly using ground spraying of residual insecticides [3]. The tsetse and trypanosomosis problem seemed to have disappeared until flies were detected again in 1998 (unpublished report of the Direction de l'Elevage - DIREL). In 2005, the DIREL initiated a control campaign called «Projet de lutte contre les glossines dans les Niayes» with the objective of developing a sustainable solution to the tsetse and trypanosomosis problem in the Niayes. The programme is funded by the Government of Senegal and technically and financially supported by the Food and Agriculture Organization of the United Nations (FAO) and the International Atomic Energy Agency (IAEA). The project is implemented in the context of the African Union - Pan African Tsetse and Trypanosomiasis Eradication Campaign (PATTEC), a political initiative of the African heads of state that calls for increased efforts to manage the tsetse and trypanosomosis problem.

Tsetse populations may be reduced using a variety of techniques, including insecticide impregnated traps and targets, live-baits, sequential aerial spraying, and sterile male releases [4]–[9]. In the past, most control efforts were not implemented according to area-wide principles [10], [11], and as a consequence, when the control effort was reduced or stopped, the tsetse populations tended to recover – due to either flies surviving the initial interventions, or migrant flies coming from untreated regions, or both [12]. This has fuelled a debate as to whether in some instances “eradication”, defined by FAO [13], [14] as the creation of a tsetse free zone, may be more cost effective than “suppression” where tsetse densities are reduced to a level minimizing the risk of disease transmission. A sound decision whether to select an eradication or suppression strategy will be facilitated when the population structure within the target region, in particular the degree of genetic isolation of the target population from its adjacent populations is clearly understood. For isolated populations, eradication may be the most cost-effective strategy, as reported for *Glossina austeni* Newstead in Unguja Island, Zanzibar [5]. But for most mainland populations of tsetse, the geographical limits of target tsetse populations are less easily defined. Application of population genetics techniques can quantify rates of gene flow between sub-populations [15]–[19], and guide decisions on the choice of control strategies [20].

**Figure 1:**
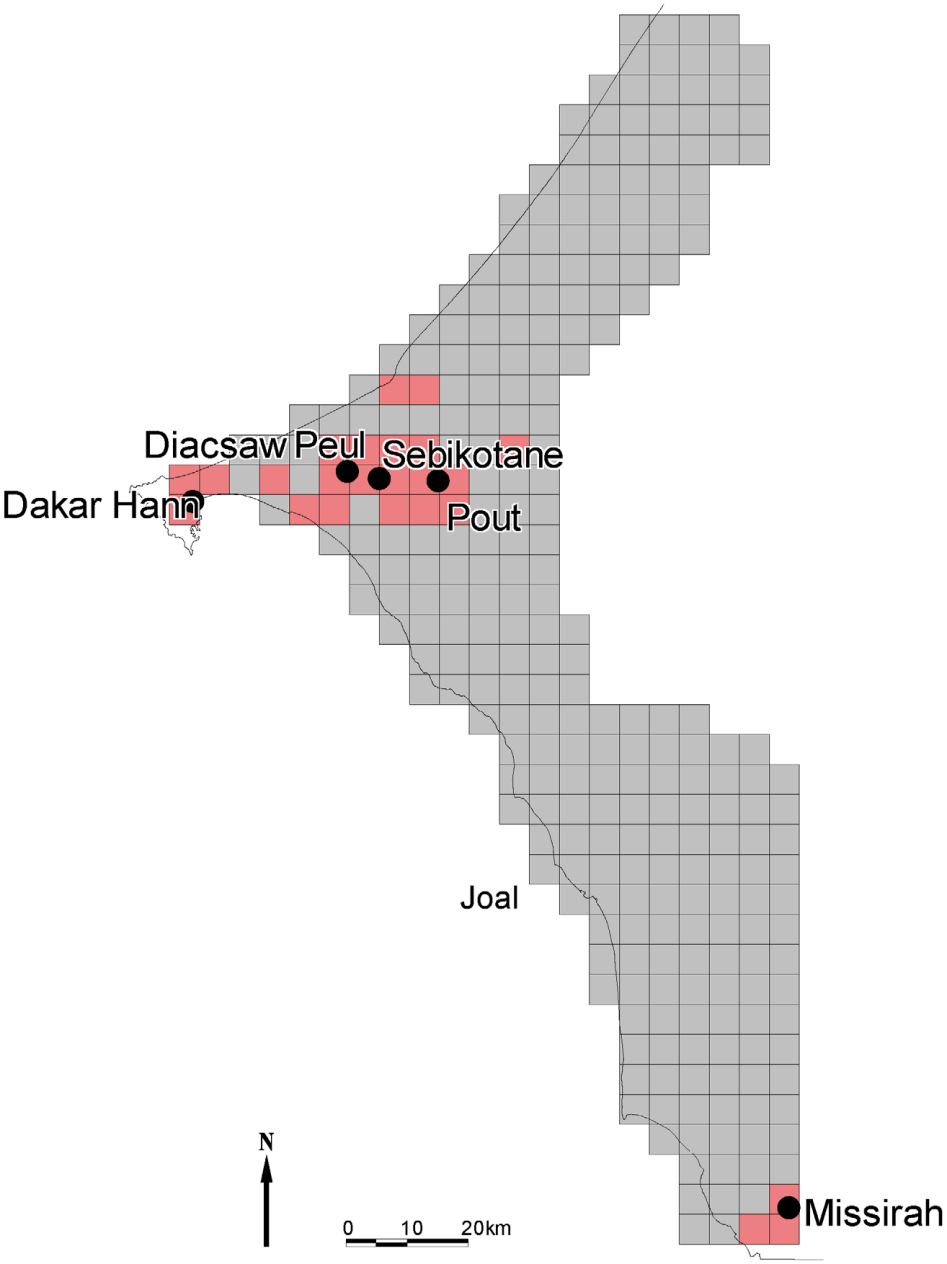
Geographic location of the study sites in Senegal. The study sites are located on the map, together with putative tsetse-infested area (red cells), according to the baseline entomological data collection of the tsetse eradication campaign in the Niayes of Senegal.

The level of isolation of the targeted tsetse populations will be an important parameter to guide the Government of Senegal to select the most optimal control strategy. Here we report population genetic analyses of microsatellite and mtDNA markers combined with morphometrics of *G. p. gambiensis* populations sampled from the Niayes area and from the nearest population in the south-eastern part of the country (fig. 1) to assess their degree of isolation by measuring gene flow among the different populations. The genetic differentiation of the various *G. p. gambiensis* populations within the Niayes was also assessed to determine if the different populations of the Niayes can be targeted at the same time (if it is a single panmictic unit), or if a sequential control strategy can be contemplated (if substantial genetic differentiation between populations is found)., which will also depend on their respective history, including effective population sizes and possible bottlenecks.

## Methods

### Study sites and sampling

In the Niayes region, four tsetse populations were sampled using Vavoua traps: Dakar Hann which is a swamp forest harbouring an animal park within the city of Dakar, Diacsaw Peul, an area of riparian thicket where tsetse and cattle are in intense contact, Sebikotan and Pout, which are mango and citrus-tree plantations where tsetse and people are in close contact. The tsetse flies collected in the Niaye area and analyzed in the present study are the sole property of the Senegalese authorities. They were collected by the national veterinary services through official mission orders, in one wildlife park (Parc de Hann) and three private sites, with the oral consent of the owners. No written consent is mandatory for tsetse fly collection in Senegal. In the south-eastern part of the country, the area of Missira was sampled: it is the nearest known infested area from the Niayes, according to a detailed tsetse survey implemented as part of the baseline data collection of the Niayes tsetse Control project (see Fig. 1). Areas between the Niayes and Missira are not favourable for tsetse, which was confirmed by zero tsetse catches (JB, BS unpublished data) despite intensive trapping efforts.

In total 153 tsetse individuals were analysed originating from Diacsaw Peul (22 females (F), 8 males (M)), Dakar Hann (23F, 6M), Sebikotan (21F, 11M), Missira (23F, 12M), and Pout (13F, 14M).

### Microsatellite DNA markers

#### Markers used and PCR conditions

A total of 10 microsatellite markers were used (preceded by “X” for X-linked loci): X55.3 [21], XPgp11, Pgp1, XPgp13, Pgp24 [22], A10, XB104, XB110, C102 (A. Robinson, FAO/IAEA, pers. com.) and GPCAG [23]. The samples were then processed for Polymerase Chain Reaction (PCR) and genotyping on a 4300 DNA Analysis System from LI-COR (Lincoln, NE) exactly as described in [19].

#### Population structure analyses

Wright's *F*-statistics [24], the parameters most widely used to describe population structure [25], were initially defined for a three-levels hierarchical population structure (individuals, sub-populations and total). In such a structure, three fixation indices or *F*-statistics can be defined. *F*_IS_ is a measure of the inbreeding of individuals (hence I) resulting from non random union of gametes within each sub-population (hence S). *F*_ST_ is a measure of the relatedness between individuals resulting from non-random distribution of individuals among sub-populations, relative to the total population; *F*_ST_ quantifies the differentiation between sub-populations in the total population (hence S and T). *F*_IT_ is a measure of the inbreeding of individuals resulting both from non-random union of gametes within sub-populations and from population structure (deviation from panmixia of all individuals of the total population, hence I and T). These *F*-statistics are classically estimated by Weir and Cockerham 's unbiased estimators *f* (for *F*_IS_), *θ* (for *F*_ST_) and *F* (for *F*_IT_) [26]. When appropriate, these statistics were estimated with Fstat 2.9.3.2 (updated from [27]). However, more than two levels (i.e., individuals, sub-populations and total) may exist. This is the case for the different subsamples belonging to the Niayes region when to be compared to tsetse from Missira. Hierfstat version 0.03–2 [28] is a package for the statistical software R [29]. This package computes hierarchical *F*-statistics from any number of hierarchical levels [28]. A user-friendly description of the software is presented elsewhere [30].

The significance of the *F*-statistics was tested by randomization (10000 permutations in each case). The significance of *F*_IS_ was tested by randomizing alleles between individuals within sub-samples. The significance of *F*_ST_ was tested by randomizing individuals among sub-samples. These tests were performed with Fstat. The significance of *F*_SR_, i.e., the homozygosity due to subdivision into subsamples within the Niayes region, was tested by randomising individuals among subsamples of the Niayes. The significance of *F*_RT_, which measures the relative homozygosity due to the geographical separation between the Niayes region and Missira was tested by randomizing subsamples (with all individuals contained) between the two regions. These two last tests were performed with Hierfstat. For *F*_IS_ the statistic used was directly the *f* (unbiased estimator of *F*_IS_). For other differentiation measures (*F*_ST_, *F*_SR_, *F*_RT_), the statistic used was the maximum likelihood ratio *G* [28], [31].

Non random association of alleles within individuals (*F*_IS_>0) may be due to the presence of null alleles. We used Micro-Checker 2.2.3 [32] to detect null alleles and estimate their frequency *p_n_* at each locus according to Brookfield's second method [33]. For X-linked loci, males were not included in such analyses and null frequency was also directly estimated by the proportion of missing genotypes. The global expected frequency of blanks (null homozygotes) was then compared to the observed one with an exact unilateral (H1: there are less blanks than expected) binomial test with R.

Linkage disequilibrium between pairs of loci was tested using Fstat by randomising loci combinations (free recombination) across loci with a *G*-based statistics permitting a global test across sub-samples for each pair of loci. Because there are as many tests as locus pairs tested, we used the exact binomial test to check if the proportion of tests found significant at the 5% level was significantly above 0.05 with the “Test alpha' and look for k'” option of Multitest V.1.2 [34]. To optimise power we disregarded loci displaying one allele at a frequency above 0.9, which was the case of locus XB110.

Some loci are X-linked. For *F*_IS_ based analyses males were coded as missing data at these loci, otherwise these loci were coded as homozygous (differentiation and LD based analyses).

In order to visualize the partition in genetic differentiation among all samples, a NJTree dendrogram was constructed with the software MEGA 3.1 [35] from a chord distance matrix (noted *D_C-S&E_*) [36], as recommended by [37], and computed using Genetix V4 [38].

#### Effective population sizes

Effective population sizes were computed with three methods. Estim 1.2 [39] uses the connection between migration and effective population size with heterozygosity and linkage disequilibrium between loci. Linkage disequilibrium is indeed connected to effective population size, a property exploited by the method from Bartley et al. [40], which is in fact coming from Hill [41] and modified by Waples [42]. From equation 2 of [40], modified as in NeEstimator Help file [43], allelic correlations (*r*) can be estimated as 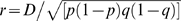, where *p* and *q* are frequencies of allele A at locus 1 and allele B at locus 2, respectively and *D* is the so called (e.g. [40], [44]) Burrow's composite measure of disequilibrium [45]. The *r* ^2^-values across all pairs of alleles are averaged to yield a single *r* ^2^ for each pair of loci. Finally, an arithmetic mean of the *r* ^2^ values for all pairs of loci is used to obtain a single correlation coefficient and to obtain an *N_e_* estimate using the equation 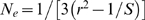 where 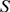 is the harmonic mean of the sample sizes of each pairwise comparison between loci [40], [46]. Males were coded homozygous for X-linked loci, which should not be a problem for a composite based linkage disequilibrium measure. The method was implemented with NeEstimator [43]. Heterozygote excess method from Pudovkin et al. [47] (see also [48]) corrected by Balloux [49], uses the fact that, in dioecious (or self incompatible) populations, alleles from females can only combine with alleles contained in males and a heterozygote excess is expected as compared to Hardy-Weinberg expectations, and this excess is proportional to the effective population size. This method was implemented using Weir and Cockerham estimator of *F*_IS_ in the equation *N_e_* = 1/(−2*F*_IS_)−*F*_IS_/(1+*F*_IS_) [49] and was only applicable in subsamples with heterozygote excess.

For intra locus based methods (Estim and Balloux's methods), loci showing evidence of stuttering and null alleles were removed.

#### Bottleneck detection

Signatures of bottleneck events were investigated by comparing the expected heterozygosity for a sample (*H_E_*) with the heterozygosity that would be expected for a sample taken in a population at mutation/drift equilibrium with the same size and allele number (*H_EQ_*). As allele number decreases faster than heterozygosity, a bottleneck is signed by *H_E_* > *H_EQ_* in subsequent generations [50]. This analysis was performed with Bottleneck v1.2 software [51] assuming that mutations of microsatellite loci followed either an IAM (infinite allele model), a SMM (stepwise mutation model), or a TPM (two phase model), in the last case of which we assumed that 70% of mutations consist of one step and 30% consist of multistep change with a variance of 30 (default values). Tests were performed using unilateral Wilcoxon tests as recommended [50].

The method of bottleneck detection described by Cornuet and Luikart [50] allows a rough estimate of the effective population size right after the bottleneck event (*N_e_*_-pb_). From the Figure 3A of [50], with 10 loci and mean sample sizes of 10–30 individuals, a bottleneck can be detected if it occurred between *τ*_1_2*N_e_*_-pb_ and *τ_2_*2*N_e_*_-pb_ generations before sampling with *τ*_1_ = 0.1 and *τ*_2_ = 2.5.

The difference in genetic diversity, as measured by *H_s_* (Nei's unbiased estimator [52]), between the Niayes and Missira was evaluated with a bilateral Wilcoxon signed rank test for paired data. The different microsatellite loci were used as the pairing factor. This test was undertaken with R.

The Bonferronni procedure [53] was used each time multiple testing was done and individual tests significance required.

User-friendly descriptions of most of the tests and procedures used in the genetic data analyses can be found in a recent review [54].

### Mitochondrial DNA markers

A portion of the 5′ end of the mitochondrial gene COI was amplified, purified and sequenced using the primers CI-J-2195 TTGATTTTTTGGTCATCCAGAAGT [55] and CULR TGAAGCTTAAATTCATTGCACTAATC using the same conditions reported by [56]. The following statistics were calculated using DNAsp version 4.50.3 [57]: *Hd* Haplotype diversity, was calculated using equations 8.4 and 8.12 in [58], *Pi*, the nucleotide diversity, which is the average number of nucleotide differences per site between two sequences, and its sampling variance was calculated using equations 10.5–10.7 in [58], *K*, the average number of nucleotide differences and the total variance of *K* is (sampling plus stochastic), assuming no recombination were calculated using equations from [59]. *F_ST_* was calculated according to equation 3 in [60], *H_ST_* was calculated according to equation 2–4, and K*_ST_ according to equations 7–11 in [60]. *H_ST_* is a haplotype frequency based genetic differentiation statistic that does not take into account the number of differences separating different haplotypes. *H_ST_* = 1−(*H_S_/H_T_*), where *H_S_* is the weighted average of subpopulation genetic diversities and *H*_T_ is the estimated haplotype diversity of the total population. K*_ST_ = 1−(*K_S_/K_T_*), where K*_S_ is a weighted average of the log corrected average number of sequence difference in the populations being compared, and *K_T_* is the average number of difference between sequences. A permutation test, in which haplotypes or sequences were randomly assigned to the different localities 10000 times, was used to test the significance of *H_ST_* and K*_ST_) [60]. The average number of nucleotide difference (equation A3, [61]) and its variance (sampling plus stochastic) were also calculated using DNAsp version 4.50.3. Haplotype trees for 738 nucleotides of *COI* from *G. p. gambiensis* (data from a total of 148 individuals in this study) were generated using the algorithm of [62]. The TCS 1.21 programme was used to estimate the haplotype tree, with the connection limit (probability of parsimony) at 95% [63]. The maximum number of connection steps at 95% was 11.

### Geometric morphometrics

In total, the number of analysed wings was 20, 21, 18, 34 and 18 for Diacsaw Peul, Dakar Hann, Sebikotan, Missira and Pout respectively. These analysed individuals were all also analysed by the molecular markers.

Wings were dry-mounted between two microscope slides and scanned with a scanner. From this picture, 10 landmarks defined by vein intersections were recorded as previously described [64]. Each landmark has X and Y coordinates, and the 10 LM defined per wing represent a polygon. After scaling, translating and rotating all these polygons so that they can be compared, data were subjected to generalized Procrustes analysis (GPA) [65], [66] allowing to implement shape variables, here represented by 16 “partial warps” (PW) (including uniform component of shape). These PW were used to conduct a discriminant analysis to allow for individual reclassification based on Mahalanobis distances (noted *D_M_*), which were calculated between populations. The statistical significance of Mahalanobis distances was estimated by 1,000-runs permutation tests [67]. A re-classification score was computed where individuals are assigned to each group and the percentage of good classification was then calculated.

### Evaluating correlation between genetic distances and morphometric distance matrices

To evaluate the correlation between distance matrices we undertook three Mantel tests [68] with the “Mantelize it” option of Fstat 2.9.4 [27] between *D_C-S&E_* and Kst*, between *D_C-S&E_* and *D_M_*, and between Kst* and *D_M_*.

## Results

### Microsatellite loci

#### Within subsamples analyses

The per-locus number of alleles was 3.4, 3.7, 3.9 and 4 for Diacsaw Peul, Dakar Hann, Sebikotan and Pout respectively (all coming from the Niayes), but it was 8.7 for Missira. The Wilcoxon test confirmed that mean genetic diversity in the Niayes (*H_s_* = 0.5) was lower than the one found in Missira (*H_s_* = 0.7) (*P*-value = 0.0059).

**Figure 2:**
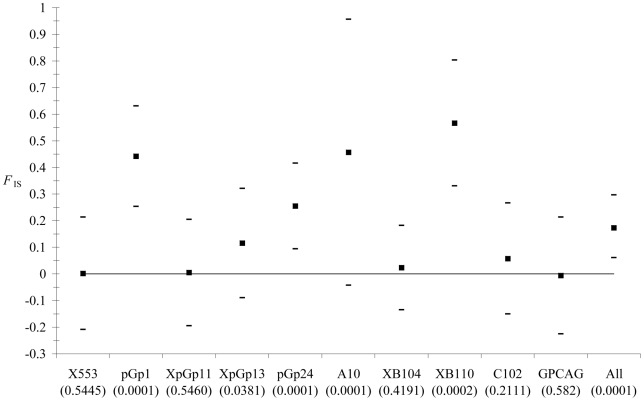
*F*_IS_ across loci and overall loci. For each locus 95% confidence intervals (CI) were obtained by jacknives over populations. Overall loci (All), CI were obtained by bootstrap over loci. Deviations from panmixia are given as corresponding *P*-values between brackets.

There was a global and significant homozygote excess over all loci (*F*_IS_ = 0.17, *P*-value = 0.0001). Figure 2 shows that this is mainly due to certain loci (i.e. pGp1, pGp24, A10 and XB110). This strongly suggests locus specific technical problems. Microchecker analysis confirmed this view as only A10 and XB110 did not display enough blanks (not enough male blanks for XB110) as compared to expected ones if *F*_IS_ were only due to null alleles. However, both loci seemed to suffer from stuttering. Without these four loci, the six remaining loci did not result in rejection of the hypothesis of panmixia (*F*_IS_ = 0.035, *P*-value = 0.0983).

Among the 36 possible tests, four pairs of loci displayed a significant linkage at the 5% level of significance. This proportion (4/36) is not different from the proportion expected under the null hypothesis (exact binomial test, *P*-value = 0.1037).

#### Hierarchical population structure

**Figure 3:**
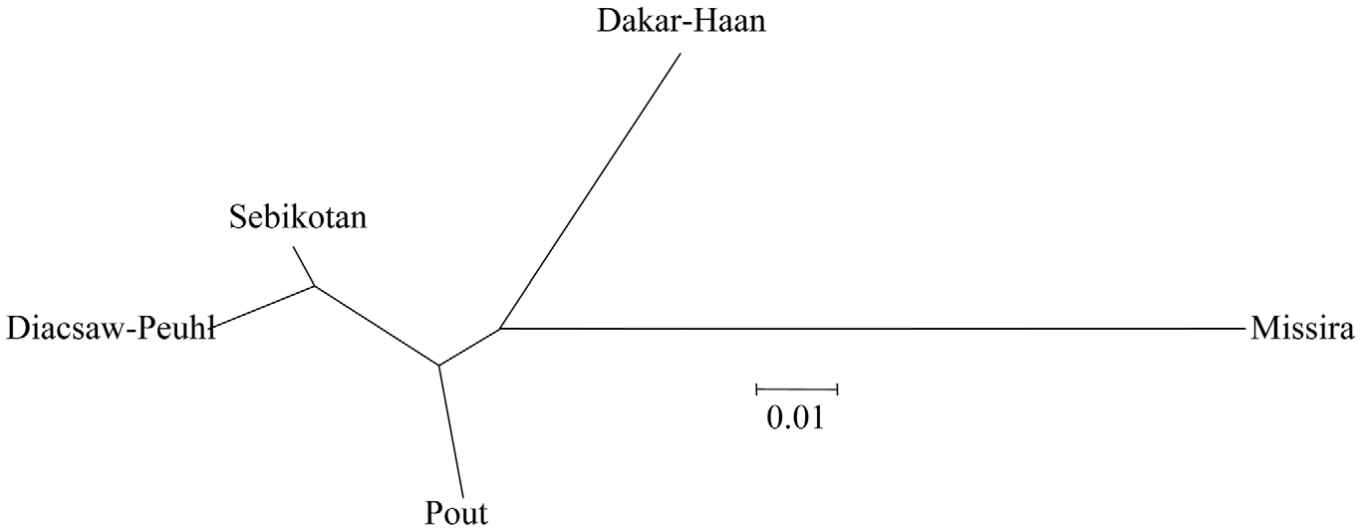
Unrooted Cavalli-Sforza and Edward's chord distance based Neighbour-Joining-Tree. This tree shows the respective genetic distribution of the five *G. palpalis gambiensis* tsetse samples from Senegal.

The overall mean *F*_ST_ value was estimated at *θ* = 0.11 (CI_95_: 0.088<θ<0.143). The mean *θ* value between Missira and all the Niayes populations was high and significant, θ = 0.12 (CI_95_: 0.08<*θ*<0.14, p<0.001). Within the Niayes, the mean *θ* value between Dakar Hann and the three others was also high and significant *θ* = 0.12 (CI_95_: 0.05<θ<0.20, p<0.001), which is of the same order of magnitude as the one between Missira and the Niayes. This is illustrated in Fig. 3, and this explains why HierFstat analysis reveals a substantial and highly significant contribution of sites (*F*_SR_ = 0.069, *P*-value = 0.001) and an absence of significant contribution of region (*P*-value = 0.209). This latter point is due to the fact that Dakar-Hann is as different from the other Niayes subsamples, than Niayes are from the remote Missira subsample.

#### Effective population sizes

A constant seemed to be that Diacsaw Peul, Dakar-Hann and Sebikotan displayed very small effective population sizes (3 to 28), and Pout and Missira appeared much bigger (51, 83 respectively). However, some of the computations led to infinite population sizes and sometimes the different methods did not converge to a reliable estimator (see Table S1), which suggests bigger effective population sizes than those found.

#### Bottleneck

Only two subsamples provided some evidence for a bottleneck: Dakar-Hann with *P*-values of 0.001, 0.001 and 0.116 for IAM, TPM and SMM mutation models respectively; and Pout with *P*-values 0.012, 0.348 and 0.935 for IAM, TPM and SMM (see Table S2). These values were still significant after Bonferronni procedure for multiple testing in Dakar Hann only: *P* = 0.015 and 0.022 for IAM and TPM respectively. It is thus probable that a bottleneck affected the population from Dakar-Hann, between 0.2*N_e_*_-pb_ and 5*N_e_*_-pb_ generations ago. Using 16 as a proxy for the effective population size in this site (obtained with Bartley's method) ranges the bottleneck between 3.2 and 80 generations ago. With the 95% confidence interval upper limits (i.e. 23) the ranges increases to [5–115]. This bottleneck provides a good explanation for the genetic isolation noticed for this site during the population differentiation analysis.

### Mitochondrial DNA

**Figure 4:**
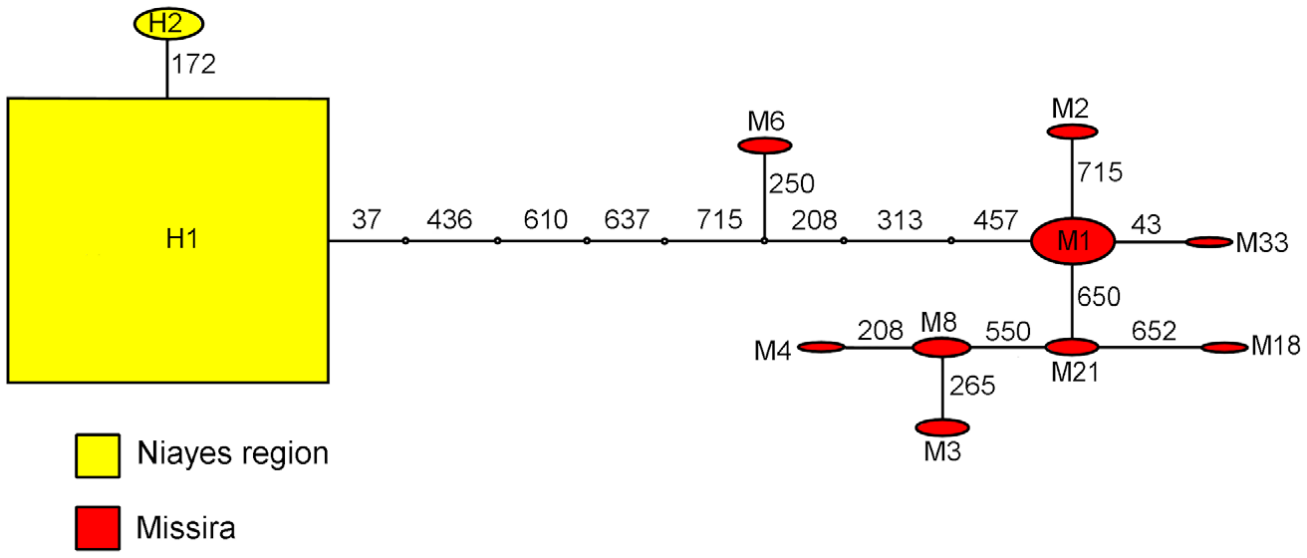
Haplotype trees for 738 nucleotides of COI from *G. p. gambiensis*. Nodes are labelled with the name of one of the individuals having a haplotype at that node. All the Niayes haplotypes are included in the nodes labelled H1 and H2. Node size is proportional to the number of haplotypes at that node. Abbreviations: H: Dakar Hann, M: Missira. The numbers on the branches indicate the position of the nucleotide in the alignment which is mutated.

The statistics of genetic differentiation between the Niayes population samples from Sebikotan, Pout, Dakar Hann and Diasca Peuhl and the south-eastern Senegal site Missira based on 738 nucleotides of mitochondrial *cytochrome oxidase 1* (*COI*) are shown in table 1. Statistics of genetic differentiation based on nucleotide sequence and haplotype frequency both indicate a very high level of genetic differentiation between Niayes populations and Missira, since there were no common haplotypes between the two regions (see Fig 4). Genetic differentiation between Diacsaw Peul and Pout was also significant (0.05>P>0.01). Otherwise, there was no significant evidence for differentiation between populations within the Niayes region. The haplotype and nucleotide diversity of the Niayes populations was very low in all cases (see table 2).

**Table 1:** Population differentiation statistics for mitochondrial *cytochrome oxidase 1* (*COI*) sequences (738 sites in alignment, no gaps).

Population	Population	Hs	Fst	Kst*	Hst
Missira	Sebikotan	0.431	0.88	**0.642**	**0.405**
Missira	Diacsaw Peul	0.415	0.883	**0.646**	**0.426**
Missira	Dakar Hann	0.499	0.876	**0.607**	**0.350**
Missira	Pout	0.611	0.865	**0.561**	**0.253**
Sebikotan	Diacsaw Peul	0.032	0.000	−0.002 ns	−0.002 ns
Sebikotan	Dakar Hann	0.099	−0.02	−0.007 ns	−0.007 ns
Sebikotan	Pout	0.200	0.137	0.087 ns	0.087 ns
Diacsaw Peul	Dakar Hann	0.067	0.038	0.022 ns	0.025 ns
Diacsaw Peul	Pout	0.171	0.208	0.134 (0.05>P>0.01)	0.134 (0.05>P>0.01)
Dakar Hann	Pout	0.256	0.062	0.035 ns	0.035 ns

Bold type indicates significant tests (P<0.001) after sequential Bonferroni correction for multiple testing.

**Table 2:** Diversity statistics for the studied populations based on *COI* sequences.

Population	Number of individuals sequenced	Number of Haplotypes	Polymorphic Sites	Hd	Pi	K (variance)
Missira	34	9	10	0.777 (3.87×10−3)	0.00271 (2×10−7)	1.996 (1.333)
Sebikotan	32	2	1	0.063 (3.33×10−3)	0.00008 (0×10−7)	0.063 (0.019)
Diacsaw Peul	30	1	0	0.000 (0.0000)	0.00000 (0×10−7)	0.000 (0.000)
Dakar Hann	27	2	1	0.142 (7.43×10−3)	0.00019 (0×10−7)	0.142 (0.045)
Pout	25	2	1	0.380 (8.33×10−3)	0.00051 (0×10−7)	0.380 (0.139)
Total	148	11	15	0.483 (2.39×10^−3^)	0.00441 (2×10^−7^)	3.257 (2.849)

### Morphometrics

An analysis of the shape of the wings was conducted by using Procustes analysis to compare wing shape among the five different populations. All Mahalanobis distances between populations were significant except between Pout and Sebikotan. This is illustrated by reclassification scores which were over 70% indicating differentiation between populations (see Table 3). Missira sample showed the best one (85%) confirming this sample was easily distinguished from the others.

**Table 3:** Geometric morphometrics: reclassification scores of individuals in their population of origin.

	Proportion of individuals correctly reclassified in their population (%)
Diacsaw Peul	15/20 (75)
Dakar Hann	16/21 (76)
Sebikotan	14/18 (77)
Missira	29/34 (85)
Pout	13/18 (72)

### Correlation between distance matrices

**Figure 5:**
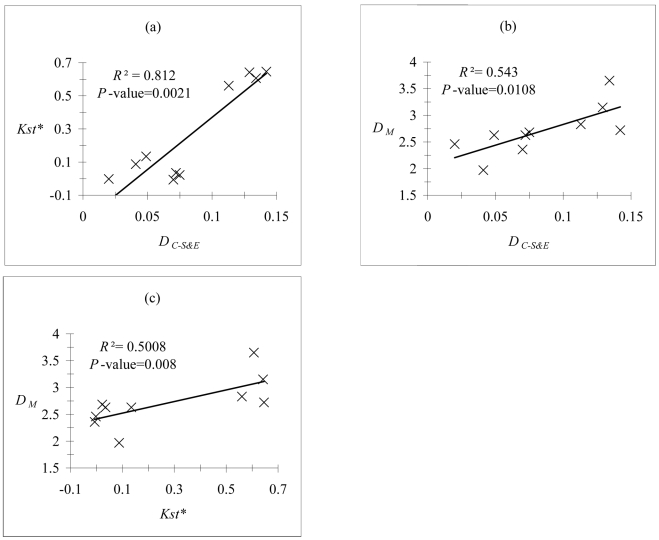
Mantel correlation tests (*P*-values) between the different markers used in this study. Mantel correlation tests and percentages of variance explained (*R* ^2^) between (a) Cavalli-Sforza and Edwards (*D_C-S&E_*) computed from microsatellite data and Kst* computed from COI mitochondrial data, (b) *D_C-S&E_* and Mahalanobis distance (*D*_M_) computed from morphometric data, and (c) Kst* and *D*_M_, for tsetse flies from the five different samples from Senegal (10 distances).

The correlation between the genetic distances and morphometrric distances were all strong and significant (see Figure 5). The strong correlation between microsatellites and COI distance matrices was mainly due to the fact that there are two sets of points: those from the Niayes sites and those differentiating the Niayes from Missira (very strong distances for both markers). For the correlation between genetic and morphometric data, Figure 5 shows that around 50% of the variance in Mahalanobis distance can be explained by genetics, meaning that the other 50% are probably explained by environmental parameters.

## Discussion

The results of the genetic and morphometric analyses indicated limited gene flow between the *G. p. gambiensis* population of the Niayes and those of the main tsetse belt in the south-eastern part of Senegal. Using three different kinds of markers, i.e. microsatellite DNA, mitochondrial DNA and geometric morphometrics, the data led to the conclusion that the *G. p. gambiensis* population from the Niayes can be considered isolated with very little risk of re-invasion should the population eventually be eradicated. These data corroborate the results of the entomological baseline data collection and observations in the field indicating the absence of *G. p. gambiensis* in the 120 km long area between Missira and the Niayes (JB, BS unpublished results.). On this basis, eradication of *G. p. gambiensis* from the Niayes can therefore be recommended as an appropriate control strategy. The data from this study, which was part of a comprehensive baseline data collection effort, confirmed that eradication can be recommended as an appropriate control strategy, and as such the study greatly assisted in the decision making on which strategy to select. Although the isolation of the target population is not an absolute prerequisite for AW-IPM, tackling a continuous pest populations is actually more complex, requiring more resources and a long-term, regional commitment (for details, see [10], [11], [14].

Looking at *F*_ST_ values reported in previous studies on *G. p. gambiensis*, the one observed in the present work between Missira and the Niayes populations was ten times higher than those observed along 260 km on the Mouhoun river in Burkina Faso [17], and two times higher than the values observed in Guinea between Loos islands and the continent [64]. These values are of the same order of magnitude as those observed between the two different taxa, *G. p. gambiensis* and *G. palpalis palpalis* (DK, PS, unpublished data). It is also noteworthy that some of the microsatellite loci used on the individuals in this study amplified poorly, a behaviour not recorded in earlier studies of *G. p. gambiensis* [17], [19], [64]. This may be an additional argument for genetic divergence, since it is known that in different taxa polymorphisms in sequences flanking the microsatellite may occur [69] leading then to mismatches in the primer binding sites. If gene flow between the sites occurs then shared haplotypes of the mitochondrial gene COI would be expected. In fact, we observed no haplotypes shared between Missira and the Niayes region. Future work should include more detailed examination of potential cryptic species within the distribution area of *G. p. gambiensis* since such cryptic species have been suspected and shown to exist in closely related taxa [70], [71].

In case the Government of Senegal decides to integrate the sterile insect technique (SIT) as part of the AW-IPM (area-wide integrated pest management) approach, it would be advisable that prior to the release of sterile males, experiments be conducted to assess mating compatibility between the *G. p. gambiensis* from the Niayes and those which should be released if they do not originate from the same area (e.g. from Burkina Faso). Although previous studies with *G. p. gambiensis* originating from Mali and Burkina Faso revealed no mating barriers between these populations (G. Mutika, personal communication), nor did another study between *G. p. gambiensis* and *G. p. palpalis* [72], these tests would provide evidence that genetic differentiation of the Niayes population has not been accompanied by pre-mating barriers, that would threaten the success of the programme. Similarly, it may also be recommended to release sterile flies coming from Burkina Faso in the Niayes to assess their behaviour and performance (dispersal, dispersion, mobility, lifespan, mating frequency etc.) in the natural habitat. Indeed, the particular eco-system of the Niayes, the fragmented nature of the preferred vegetation types of *G. p. gambiensis* (mango-tree plantations, *Euphorbia spp.* hedges) and an annual precipitation below 400–500 mm might have induced the development of some level of xerotolerance of these relatively small populations [73], [74].

The second question that has relevance for a future AW-IPM programme was to know whether within the Niayes region, the four populations showed any genetic differentiation, or if they constituted a single, panmictic (i.e random mating) population. Low haplotype diversity of the populations of this area is in agreement with a previous Single Strand Conformation Polymorphism-based study on mitochondrial DNA haplotypes in the Niayes region of Senegal [75]. The Niayes population is probably a remnant population, possibly of small size and therefore likely to lose rare haplotypes by genetic drift more rapidly than larger populations. Many ancestral *G. p. gambiensis* haplotypes have probably been lost from the Niayes region, leaving just two haplotypes remaining today. The low diversity was also observed at microsatellite loci with lower genetic diversity than in Missira. The three different markers used in this study generally showed good agreement in the differentiations observed, as can be seen by the high and significant correlations coefficients computed between them.

The only apparent discrepancy between microsatellites on one hand and COI based and morphometrics based distances on the other hand probably comes from the variance expected for paired *F*_ST_ estimates and the small sample sizes, the effect of which is probably increased by historical events such as the probable bottleneck that affected the Dakar-Hann population. This may explain why only microsatellite data suggest that the population from Dakar Hann is so much differentiated from all the others, including the geographically closest ones. From an operational point of view however, the population of Dakar Hann is probably also isolated from the three others, since the bottleneck signature was still visible, suggesting very limited exchanges (if any) with the other populations. Field data also corroborate this since the population of Dakar Hann is located within an animal park of this huge city which can be seen as an isolated refuge for this tsetse population. This further suggests that an elimination operation may probably be implemented on this population.

The results also suggest that the samples from Diacsaw Peul, Sebikotane and Pout, although showing some genetic differentiation, are not completely isolated from each other. This is also consistent with ecological data since it appears that the maximum distance between the forest patches of this area is less than 2km (data not shown.).

To conclude, the use of genetic and morphometric markers has been instrumental in the decision-making process of selecting and developing of an appropriate intervention strategy to create a sustainably zone free of *G. p. gambiensis* and Trypanosomosis in the Niayes region of Senegal. In the near future, it should be encouraged to carry out such studies prior to the selection of target areas or the choice of control strategies, and these should be part of the overall collection of baseline data (see ref [76] for recent review). In addition, the results obtained here suggest that efforts should be made to look for other genetic discontinuities in *G. palpalis* s.l. distribution that may be indicative of the presence of cryptic species.

## Supporting Information

Alternative Language Abstract S1 Translation of the abstract into French by PS.
(0.03 MB DOC)

Table S1 Effective population size estimates according to the different methods described.
(0.02 MB XLS)

Table S2 Probabilities of detecting bottleneck in each population according to microsatellite mutation model.
(0.02 MB XLS)
