# Supplementary material for: Population Genetics as a Tool to Select Tsetse Control Strategies: Suppression or Eradication of Glossina palpalis gambiensis in the Niayes of Senegal
Source: PLoS Negl Trop Dis. 2010 May 25;4(5):e692. doi: 10.1371/journal.pntd.0000692 (PMC2876113; doi:10.1371/journal.pntd.0000692)
Supplement: Alternative Language Abstract S1 — Translation of the abstract into French by PS. (0.03 MB DOC) [file pntd.0000692.s001.doc]

Résumé

Le Gouvernement du Sénégal a initié « le projet de lutte contre les glossines dans les Niayes » afin de se débarasser de la contrainte trypanosomienne de manière durable dans cette région. Suite à des échecs de ce type de lutte anti-vectorielle dans le passé, il est apparu important de savoir si la lutte devait se baser sur une stratégie « d’éradication », ou de « suppression ». Afin de donner des éléments de réponse, nous avons mesuré la différenciation génétique entre les glossines des Niayes et celles les plus proches, situées au sud du pays (région de Missira).

Nous avons utilisé 3 marqueurs différents, des marqueurs moléculaires (ADN microsatellite et mitochondrial) et morphométriques (morphométrie géométrique des ailes) sur 153 tsé-tsé. Ils concordent pour diagnostiquer un isolement des tsé-tsé des Niayes par rapport à celles de Missira. La différenciation génétique mesurée (θ=0.012) est équivalente à celle existant entre deux taxons différents. De plus, au sein même de la région des Niayes, la population présente au sein de la ville de Dakar (Dakar Haan) semble isolée de ces voisines, et l’analyse de sa structure montre qu’elle a probablement connu un « goulot d’étranglement ».

Cette étude montre qu’une stratégie d’éradication (qui vise à éliminer toutes les tsé-tsé de la région) des tsé-tsé des Niayes peut être recommandée. Nous proposons que ce type d’étude puisse être mené dans d’autres régions d’Afrique et sur d’autres espèces de tsé-tsé pour aider les programmes de lutte à élaborer leur stratégie.
